# Deletion of Fibrinogen-like Protein 2 (FGL-2), a Novel CD4^+^ CD25^+^ Treg Effector Molecule, Leads to Improved Control of *Echinococcus multilocularis* Infection in Mice

**DOI:** 10.1371/journal.pntd.0003755

**Published:** 2015-05-08

**Authors:** Junhua Wang, Dominique A. Vuitton, Norbert Müller, Andrew Hemphill, Markus Spiliotis, Oleg Blagosklonov, Denis Grandgirard, Stephen L. Leib, Itay Shalev, Gary Levy, Xiaomei Lu, Renyong Lin, Hao Wen, Bruno Gottstein

**Affiliations:** 1 Institute of Parasitology, University of Bern, Bern, Switzerland; 2 State Key Lab Incubation Base of Xinjiang Major Diseases Research (2010DS890294) and Xinjiang Key Laboratory of Echinococcosis, First Affiliated Hospital of Xinjiang Medical University, Urumqi, Xinjiang, China; 3 Department of Nuclear Medicine, University of Franche-Comté and Jean Minjoz University Hospital, Besançon, Franche-Comté, France; 4 World Health Organization (WHO)-Collaborating Centre for the Prevention and Treatment of Human Echinococcosis, University of Franche-Comté and University Hospital, Besançon, Franche-Comté, France; 5 Neuroinfection Laboratory, Institute for Infectious Diseases, University of Bern, Bern, Switzerland; 6 Biology Division, Spiez Laboratory, Federal Office for Civil Protection (FOCP), Spiez, Switzerland; 7 University of Toronto Transplantation Institute, Toronto, Ontario, Canada; Universidad Nacional Autónoma de México, MEXICO

## Abstract

**Background:**

The growth potential of the tumor-like *Echinococcus multilocularis* metacestode (causing alveolar echinococcosis, AE) is directly linked to the nature/function of the periparasitic host immune-mediated processes. We previously showed that Fibrinogen-like-protein 2 (FGL2), a novel CD4^+^CD25^+^ Treg effector molecule, was over-expressed in the liver of mice experimentally infected with *E*. *multilocularis*. However, little is known about its contribution to the control of this chronic helminth infection.

**Methods/Findings:**

Key parameters for infection outcome in *E*. *multilocularis*-infected *fgl2^-/-^* (AE-*fgl2^-/-^*) and wild type (AE-WT) mice at 1 and 4 month(s) post-infection were (i) parasite load (i. e. wet weight of parasitic metacestode tissue), and (ii) parasite cell proliferation as assessed by determining *E*. *multilocularis* 14-3-3 gene expression levels. Serum FGL2 levels were measured by ELISA. Spleen cells cultured with ConA for 48h or with *E*. *multilocularis* Vesicle Fluid (VF) for 96h were analyzed *ex-vivo* and *in-vitro*. In addition, spleen cells from non-infected WT mice were cultured with rFGL2/anti-FGL2 or rIL-17A/anti-IL-17A for further functional studies. For Treg-immune-suppression-assays, purified CD4^+^CD25^+^ Treg suspensions were incubated with CD4^+^ effector T cells in the presence of ConA and irradiated spleen cells as APCs. Flow cytometry and qRT-PCR were used to assess Treg, Th17-, Th1-, Th2-type immune responses and maturation of dendritic cells. We showed that AE-*fgl2^-/-^* mice exhibited (as compared to AE-WT-animals) (a) a significantly lower parasite load with reduced proliferation activity, (b) an increased T cell proliferative response to ConA, (c) reduced Treg numbers and function, and (d) a persistent capacity of Th1 polarization and DC maturation.

**Conclusions:**

FGL2 appears as one of the key players in immune regulatory processes favoring metacestode survival by promoting Treg cell activity and IL-17A production that contributes to FGL2-regulation. Prospectively, targeting FGL2 could be an option to develop an immunotherapy against AE and other chronic parasitic diseases.

## Author Summary

In larval *E*. *multilocularis* infection causing alveolar echinococcosis (AE) in humans as well as mice, immune tolerance and/or down-regulation of protective immunity is a marked characteristic of this chronic disease. Our study provides a comprehensive evidence for a major involvement of the recently identified CD4^+^ CD25^+^ Regulatory T Cell Effector Molecule FGL2 to the outcome of AE. Our major findings are as follows: 1) FGL2 is mostly secreted by Tregs and partly contributes to their functions; 2) FGL2 can down-regulate the maturation of DCs, suppress Th1 and Th17 immune responses, and support Th2 and Treg immune responses, and finally 3) IL-17A contributes to FGL2 secretion. Based on the present findings in mice, we will investigate FGL2 as a potential marker of progression of AE in human patients, or as a potential immunotherapeutical target. Early prediction of parasite regression (currently not yet possible) would allow clinicians to plan for withdrawing benzimidazole treatment, which is currently administered for life. Then, FGL2 should be investigated as a target for an anticipated immunomodulatory treatment of patients with progressive AE, especially of those who are non- or low-responders to benzimidazole treatment, or who suffer from side-effects due to chemotherapy.

## Introduction

Alveolar echinococcosis (AE) is a very severe zoonotic helminthic disease in humans, exhibiting a fatal outcome if remaining untreated [1]. AE is characterized by chronic and progressive hepatic damage caused by the continuous proliferation of the larval stage (metacestode) of *Echinococcus multilocularis* [2], that behaves like a slowly growing liver cancer, progressively invading host tissues and organs [3].

During *E*. *multilocularis* infections in humans, a Th2-oriented immunity is basically associated with increased susceptibility to disease leading to chronic AE, while Th1 cell activation has been linked to protectivity, which may even yield aborted ("died-out") forms of infection [2,3]. Experimental murine AE is characterized, as studied in spleen or lymph node cells, by an initial Th1 response during the early stage of infection (till 1 month p.i.) that gradually switches to a more dominant Th2-biased response during the chronic phase of AE (2–4 months p.i.). Nevertheless, this mostly mixed Th1/Th2 profile, characterized by the concomitant presence of IL-12α, IFN-γ and IL-4 at the very early stage of *E*. *multilocularis* infection [4], is associated with the expression of pro-inflammatory cytokines in the periparasitic granuloma and partial/relative protective immunity (restriction of parasite growth) through fibrosis and necrosis [5]. It has been previously reported that CD4^+^CD25^+^ T regulatory cells (Tregs) play a critical role in human AE by blunting immune responses to specific antigens, or by suppressing the secretion of proinflammatory cytokines, especially through interleukin (IL)-10 and transforming growth factor beta1 (TGF-β1) [6]. Moreover, increased CD4^+^CD25^+^ Tregs were also observed in peritoneal cells of mice intraperitoneally (i.p.) infected with *E*. *multilocularis*, a finding that concurred with other findings demonstrating that *E*. *multilocularis* antigens promote T cell differentiation into Treg cells [7].

Previous microarray analyses showed that expression of mRNA coding for the fibrinogen-like protein 2 (FGL2) were significantly up-regulated in the liver of mice perorally infected with *E*. *multilocularis* eggs [8]. FGL2, a member of the fibrinogen-related superfamily of proteins secreted by T cells, has recently been reported by a number of groups to be highly expressed in Tregs. Its role was associated to Treg effector functions [9,10]. It was shown that FGL2 could inhibit dendritic cell (DC) maturation through binding to the low-affinity FcgammaRIIB receptor, and thus contribute to Treg activity [11]. There is evidence that FGL2 exerts an immunosuppressive effect on T cell proliferation. Thus, FGL2 seems to play an important role both in innate and adaptive immunity, by the fact to be expressed by activated CD4^+^ and CD8^+^ T cells, and reticulo-endothelial cells as well [12–17]. FGL2 has been propagated as a novel cancer biomarker, and was shown to be involved in the pathogenesis of inflammatory disorders such as allo- and xenograft rejection [12,18–22] and cytokine-induced fetal loss [23], as well as in the pathogenesis of infectious diseases, such as viral hepatitis [14,17]. However, nothing is known about FGL2 and its potential role in parasite-induced immunotolerance.

The major aims of this work were thus: 1) to study the role of FGL2 in T cell reactivity as well as its effect on the maturation of DCs in an early time-point and a late stage of *E*. *multilocularis* infection in *fgl2* knock-out (*fgl2* ^*-/-*^) mice; 2) to elucidate how parasite components, i.e. metabolites represented by those expressed in the VF of the *E*. *multilocularis* metacestode, affect the immune response in *fgl2* ^*-/-*^ mice; 3) to explore how FGL2 is secreted during the course of *E*. *multilocularis* infection; and 4) to provide a comprehensive picture of the various cell and molecular components involved in the regulation of the peritoneal periparasitic immune cell infiltrate, and likewise in the spleen as a key immune organ. To achieve these goals, Th1/Th2-related and Treg/Th17 related cytokines, the maturation of DCs, and the generation of Tregs and their functions were studied at the different disease stages in an experimental model with active or knocked-out FGL2-expression.

## Materials and Methods

### Ethics statement

The animal study was performed in strict accordance with the recommendations of the Swiss Guidelines for the Care and Use of Laboratory Animals. The protocol was approved by the Commission for Animal Experimentation of the Canton of Bern (approval no. BE_103/11).

### Mice, parasites and infection

8-week-old female C57/BL6 (wild type [WT]) and C57/BL6 *fgl2* ^-/-^ mice [24] were bred and housed in specific-pathogen-free (SPF) facilities according to recommendations of FELASA, and monitored by daily assessment of health status, putative weight loss or gain during the experiments. *E*. *multilocularis* metacestodes (clone KF5) were maintained by serial passages (vegetative transfer) in C57BL/6 mice [25] and injected intraperitoneally as previously described [26,27]. Each experimental group included 6 animals unless otherwise stated. Control mice (mock-infection) received 100 μL of RPMI-1640 only. Mice were sacrificed at 1 or 4 month(s) post-infection, corresponding to early and late stage of disease, respectively. Parasite tissues were surgically recovered and, if present, fat and connective tissues were carefully removed for subsequent wet-weight determination of the parasite mass.

### *E*. *multilocularis 14-3-3* gene-expression analyses by quantitative reverse transcriptase real time PCR (qRT-PCR)

Total RNA was extracted from parasite tissue previously put into TRIzol (Invitrogen) according to the manufacturer’s instructions. cDNA was synthesized using the Omniscript Reverse Transcription kit (Qiagen, Hilden, Germany). SYBR-Green Mix-based qRT-PCR was carried out on a Rotor-Gene 6000 qPCR detection system (Corbett) with the FastStart Essential DNA Green Master (Roche, Basel, Switzerland) following the manufacturer’s instructions. PCR cycling was performed in triplicates in final volumes of 20 μL containing 2 μL cDNA and 10 pM of each primer (Cycle scheme: initial denaturation at 95°C—15 min, 45 cycles of 95°C—15 s, 55°C—30 s and 72°C—30 s). Fluorescence was measured in every cycle, and a melting curve was analyzed after the PCR by increasing the temperature from 55 to 95°C in 0.5°C increments. The primers used were described earlier [28], and *em14-3-3* mRNA levels were quantified relative to the mRNA level of a parasite housekeeping gene, the *β-actin* homologue *E*. *multilocularis*. Respective mean values from triplicate determinations from 6 individual mice in each group were taken for the calculation of relative *emII/3* and *em14-3-3* mRNA levels in relation to *em-β-actin* mRNA levels).

### Cell preparations and Treg suppression assay

Peritoneal exudate cells (PEC) and spleen cells were collected by peritoneal rinsing or grinding in 5 mL RPMI-1640 (Gibco, Basel, Switzerland) and incubation of PEC or spleen cell suspensions in 15 mL RPMI-1640 +20%FCS in a petridish for 2 h at 37°C 5%CO_2_, as described earlier [25]. The non-adherent cells were collected, and highly (N99%) enriched iTreg cells were obtained by MACS (magnetic cell-separation) using the mouse CD4^+^CD25^+^ T cell Isolation-Kit (Miltenyi Biotec, Germany) followed by FACS.

*In vitro* suppression assays were carried out with cultures of 2×10^4^ CD4^+^CD25^-^ T effector (Teff) cells from WT-mice as responder cells, together with 8×10^4^ irradiated spleen cells as APCs and titrated numbers of CD4^+^CD25^+^ Treg cells from either *E*. *multilocularis-*infected AE-*fgl2* ^*-/-*^ or AE-WT mice as suppressor-cells, compared with non-infected controls. For rFGL2/antibody blockade studies, 1 μg/mL mouse rFGL2 or anti-FGL2- (monoclonal IgG2a; Abnova, Luzern, Switzerland) were added to the cell cultures at a 1:1 Treg:Teff ratio in the presence of APCs and ConA (2μg/mL). Cell proliferation was assayed using the colorimetric BrdU cell proliferation ELISA kit (Calbiochem, Merck, Switzerland) according to manufacturer’s instructions.

### Spleen cell stimulation assays and FGL2 measurement

Spleen cells were cultured at a density of 2×10^6^ cells/mL in RPMI-1640 +10%FCS. For assessment of the effects of stimulation by recombinant FGL2 (rFGL2) or anti-FGL2 monoclonal antibodies (anti-FGL2-MAb), (both from Sigma-Aldrich, Basel, Switzerland) they were incubated with 1 and 5 μg/mL rFGL2 or 1 μg/mL anti-FGL2-MAb for 48h in the presence of a protein transport inhibitor cocktail (Ebioscience, San Diego, CA, USA) for cytokine staining. Negative control reactions were performed without rFGL2 or without anti-FGL2-MAb. The effects of recombinant IL-17A (rIL-17A) anti-IL-17A antibodies (both from Sigma), were assessed by stimulation of cells with 0.5, 1, 2 and 4 μg/mL rIL-17A or 1 μg/mL anti-IL-17A for 48h, while negative control reactions were performed without rIL-17A or anti-IL-17A antibodies. FGL2-levels in the serum and supernatant from cell cultures were measured by sandwich ELISA (Biolegend, San Diego, CA) according to manufacturer’s instructions. Spleen cell cultures were also stimulated with 2 μg/mL ConA for 48h, or with 10μg/mL of VF for 96 h, in the presence of protein transport inhibitor cocktail for cytokine staining. The same cell reactions performed without VF were used as negative controls.

### Flow cytometry

PEC or spleen cells were incubated with 1 μg of purified anti-CD16/CD32 for 20 min in the dark to block non-specific binding of antibodies to the FcγIII/II receptors, cells were then stained with surface markers separately for 15 min with 1 μg of primary antibodies: FITC-labeled anti-CD80, anti-CD86, anti-CD25; PE-labeled anti-CD11b, anti-CD11c, PECy 5.5-labeled anti-CD4. For intracellular staining, the cells were first incubated with Inside-Fix (Miltenyi, Bergisch Gladbach, Germany) for 20 min at room temperature then stained with PE-labeled anti-IFN-r, anti-IL-4, anti-IL-17A, anti-IL-2, anti-IL-10 and anti-Foxp3 in Inside-Perm (Miltenyi, Bergisch Gladbach, Germany) for 15 min in the dark. Corresponding fluorochrome-labeled isotype control antibodies were used for staining controls. For each sample, a minimum of 500,000 cells were acquired using a FACS LSRII flow cytometer and analyzed using FlowJo software (Tree Star, OR, USA), employing the gating strategy shown in S1 Fig. All antibodies were purchased from BD Pharmingen (Palo Alto, USA).

### Statistical analyses

All data were analyzed by SPSS 17.0. The results are presented as means ± SD. Normality of data was assessed by D’Agostino & Pesrson and Shapiro-Willk test. For normally distributed groups of data, One-way-ANOVA followed by Bonferroni’s post-test or unpaired two-tail Student’s *t*-test were used to compare the differences between groups, and two-tail Spearman’s rho was used to analyze the correlation coefficient. Significance was defined as *P*<0.05 for all tests, except those subsequently corrected by Bonferroni.

## Results

### *E*. *multilocularis* infection induces FGL2 production through IL-17A

**Fig 1:**
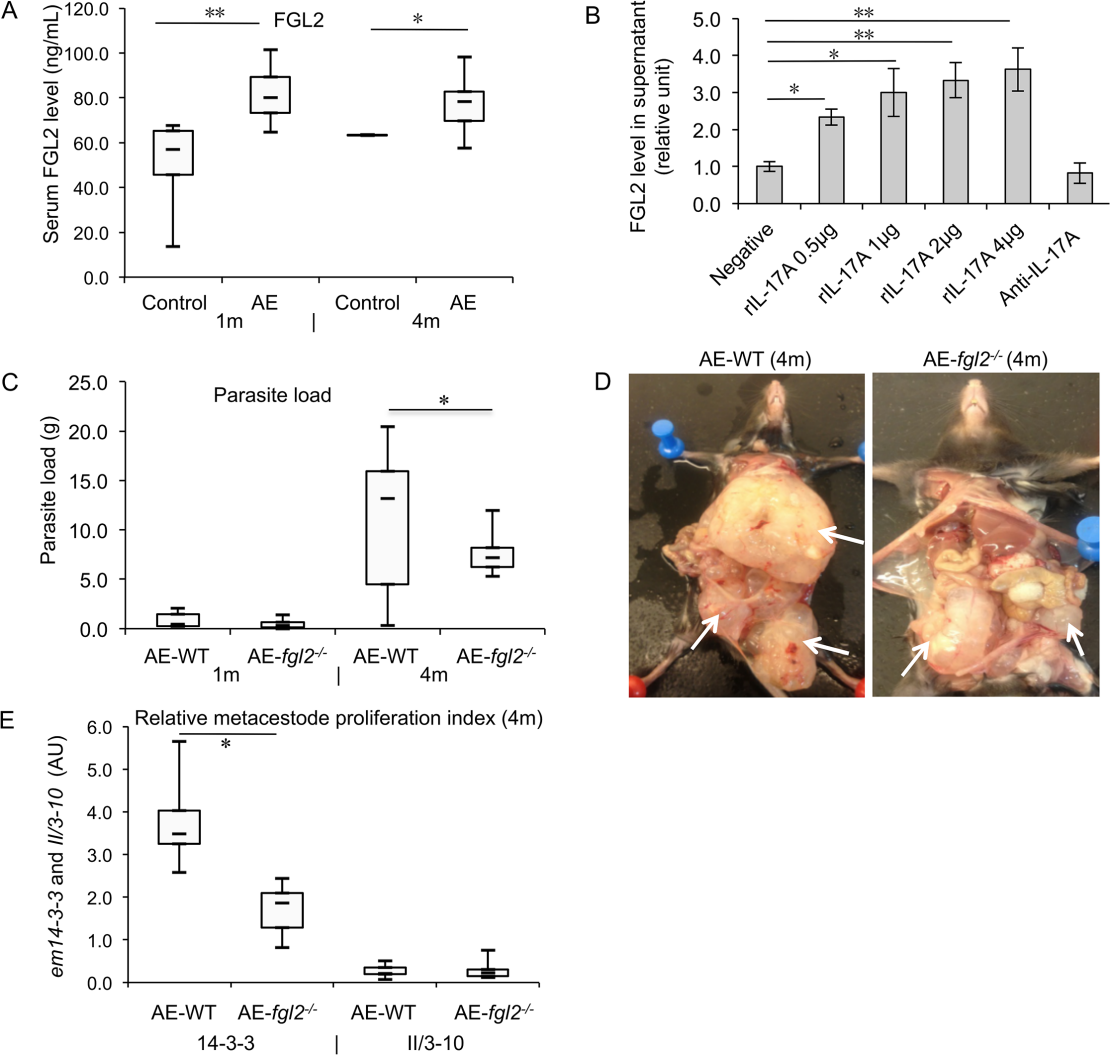
Serum FGL2 levels in *E*. *multilocularis* infected mice, effect of IL-17A on FGL2 secretion in spleen cells, and effects of FGL2 on parasite load and proliferation in *E*. *multilocularis* infected mice. (A) Serum levels of FGL2 in infected AE-WT mice at different stages of infection, as compared to non-infected control mice (Control). (B) Different concentrations of recombinant IL-17A (0, 0.5, 1, 2 μg/mL) or anti-IL-17A MAbs (1 μg/ml) were added to primary spleen cells isolated from non-infected WT mice (4 months p.i.). FGL2-levels in culture supernatants were determined by ELISA. (C) Parasite load in AE-WT and AE- *fgl2* ^*-/-*^ mice assessed by wet weight measurement at 1 month and 4 months post- infection. (D) Representative images of *E*. *multilocularis* infection in AE-WT and AE-*fgl2* ^*-/-*^ mice at 4 months p.i.; arrows point at intraperitoneal metacestode tissue/lesions. (E) Parasite cell proliferation in AE-WT versus AE-*fgl2* ^*-/-*^ assessed by *em14-3-3* qRT-PCR at 4 months p.i, upon comparison to the constitutively expressed house-keeping Em II/3-10 gene. Data represent mean ± SD of three independent experiments of a total of 15–18 mice in each group (5–6 mice per group in each independent experiment). Comparison between groups was performed using a one-way ANOVA for statistical analysis. *P<0.05, ** *P*<0.01. ‘WT’, wild type mice; ‘*fgl*2^*-/-*^’, *fgl2* knock-out mice; ‘AE-WT’, *E*. *multilocularis*-infected wild type mice; ‘AE-*fgl2* ^*-/-*^’, *E*. *multilocularis*-infected *fgl2* knock-out mice. ‘Control’, non-infected mice; ‘1m’, 1 month p.i.; ‘4m’, 4 months p.i. AU: arbitrary units. Note: Em 14-3-3 is used as a molecular marker to assess viability and growth activity of the *E*. *multilocularis* metacestode tissue, whereas Em II/3-10, so as actin, serve as constitutionally expressed house-keeping gene.

The levels of FGL2 expression in sera of *E*. *multilocularis* infected (AE-WT) mice were significantly higher, both at 1 and at 4 month(s) post-infection, when compared to non-infected WT-controls (Fig 1A). Spearman correlation coefficients indicated a positive correlation between serum IL-17A level and FGL2 (r = 0.435, *P* = 0.045) in AE-WT-mice. To examine whether IL-17A contributes to FGL2-secretion, spleen cells from non-infected WT-mice were co-cultured either with recombinant IL-17A (rIL-17A) as an external stimulus, or with anti-IL-17A antibodies, and FGL2 levels were quantitatively analyzed in respective culture supernatants by ELISA. As shown in Fig 1B, addition of rIL-17A lead to increased FGL2-secretion in a dose-dependent manner, while the addition of anti-IL-17A had no effect (Fig 1B).

### FGL2 plays a critical role in the overall infection control

To characterize the role of FGL2 in the control of parasite growth, *E*. *multilocularis*-infected *fgl2* ^*-/-*^ mice and control WT littermates were analyzed after 1 and 4 months p.i. with respect to parasite weight and the expression of *em14-3-3* as a marker for cellular proliferation activity [24]. At the late stage of infection (4 months- p.i.), *fgl2* ^*-/-*^ mice exhibited a significantly lower parasite load compared to WT mice (Fig 1C and 1D), and *14-3-3* expression levels in AE-*fgl2* ^*-/-*^ mice were significantly lower those in AE-WT mice (Fig 1E). Moreover, the parasite invaded the liver (a marker of pathogenicity) in only 33.3% of the AE-*fgl2* ^*-/-*^ mice compared to 94.4% of AE-WT mice.

### FGL2 is mostly secreted by Tregs and affects Treg function at late stage of infection

**Fig 2:**
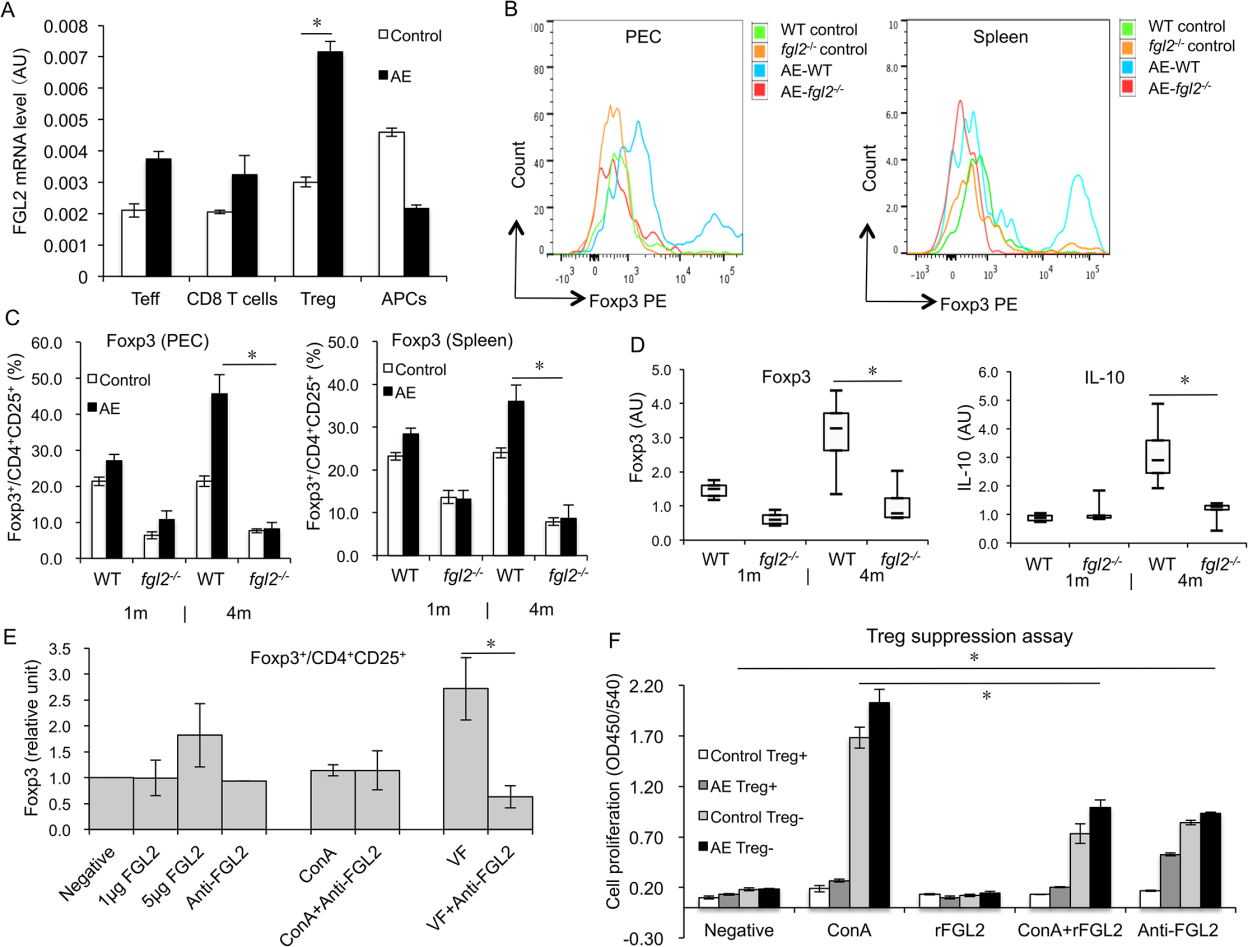
Treg secretion and function in both AE-WT and AE-*fgl2* ^*-/-*^ mice after *E*. *multilocularis* infection, and in WT mice in response to recombinant FGL2/anti-FGL2-MAb blockade. (A) Spleen was taken from non-infected WT and AE-WT mice, CD4^*+*^ Teffs, CD8^*+*^ T cells, CD4^*+*^CD25^+^ Tregs and APCs were isolated by FACS cell sorting, *fgl2* mRNA levels were determined by qRT-PCR. (B) Representative images of Foxp3 mean fluorescence intensity (MFI) from AE-WT and AE-*fgl2* ^*-/-*^ mice, and non-infected mice as controls. (C) Frequency of Foxp3^*+*^ T cells within CD4^+^CD25^+^T cells in PECs and spleen cells from AE-WT and AE-*fgl2* ^*-/-*^ mice at 1 month and 4 months post-infection. (D) Foxp3 and IL-10 gene expression in PECs during *E*. *multilocularis* infection (measured by qRT-PCR). AU: arbitrary units. Graphs show the mean±SD. Data represent mean±SD of three independent experiments of a total 15–18 mice in each group (5–6 mice per group in each independent experiment). Comparison between groups was performed using a one-way ANOVA for statistical analysis. (E) 0, 1, and 5 μg/mL of recombinant FGL2 and 1μg/mL of anti-FGL2-MAb were added to primary spleen cells from non-infected WT mice, or spleen cells stimulated with ConA or vesicle fluid (VF). Relative expression levels of Foxp3^+^/CD4^+^CD25^+^ were determined by flow cytometry. (F) CD4^+^CD25^+^ Tregs (suppressor cells) and CD4^+^CD25^*-*^ Teff cells (responder cells) were isolated from spleen cells of both non-infected and infected AE-WT mice by FACS. The two cell populations were co-cultured at a ratio of 1:1 (suppressor: responder) in the presence of APCs and ConA (2 μg/mL), rFGL2 (1 μg/mL), ConA (2 μg/mL) + rFGL2 (1 μg/mL), or anti-FGL2-MAb (1 μg/mL); cell proliferation was measured using BrdU ELISA. Data represent mean±SD of three independent experiments of a total of 15–18 mice in each group (5–6 mice per group in each independent experiment). Expression of Foxp3^*+*^/CD4+CD25^*+*^ was normalized with negative control (cells without rFGL2 and anti-FGL2-MAb (negative control) were considered as base line, e.g. as 1.0). Comparison between groups was performed using a one-way ANOVA with Bonferroni’s multiple comparison post-test for statistical analysis. *P<0.025 for *ex vivo* assay, and *P<0.006 for *in vitro* assay. ‘WT’, wild type mice; ‘*fgl2* ^*-/-*^’, fgl2 knock-out mice; ‘AE-WT’, *E*. *multilocularis*-infected wild type mice; ‘AE-*fgl2* ^*-/-*^’, *E*. *multilocularis*-infected *fgl2* knock-out mice. ‘PEC’, peritoneal exudate cells; ‘Spleen’, spleen cells; VF, vesicle fluid.

To explore the cellular source of secreted FGL2, CD4^+^ effector T cells (Teffs), CD8^+^ T cells, CD4^+^CD25^+^ Tregs, antigen presenting cells (APCs) were FACS sorted from spleen cell suspensions of AE-WT mice, and non-infected control mice. Quantitative RT-PCR showed that *fgl2* mRNA-levels were significantly increased in CD4^+^CD25^+^ Tregs derived from AE-WT mice, when compared to non-infected controls, while no significant changes were evident with respect to CD4^+^ Teffs, CD8^+^ T cells. A slight decrease in FGL2 mRNA-levels were noted in APCs from AE-WT mice compared to those in non-infected mice (Fig 2A).

The contribution of FGL2 in the generation and maintenance of Tregs in AE-WT and AE-*fgl2* ^*-/-*^ mice was analyzed *ex vivo*, and *in vitro* by either addition of rFGL2 to spleen cells or treating cultures with anti-FGL2 antibodies. At 4 months p.i., an increased frequency of Foxp3^+^/CD4^+^ CD25^+^ cells could be observed in PECs as well as spleen cells from AE-WT mice, compared to respective preparations in AE-*fgl2* ^*-/-*^ mice (*P*<0.05) (Fig 2B and 2C). In addition, expression levels of Foxp3 and IL-10-transcripts were significantly increased in PECs from AE-WT mice (Fig 2D). Moreover, when in *vitro* cultured PECs from AE-WT mice were exposed to anti-FGL2-MAbs and stimulated with VF, the frequency of CD4^+^CD25^+^Foxp3^+^ cells was decreased compared to PECs cultured in the absence of anti-FGL2-MAbs (Fig 2E). This indicated that in the absence of FGL2 *E*. *multilocularis* metabolic components might exert immune-modulatory activities.

We then assessed the effect of the targeted deletion of *fgl2* on the ability of Treg cells to suppress the proliferation of Teffs. Treg cells from either non-infected or AE-*fgl2* ^*-/-*^ mice, were less efficient in suppressing normal CD4^+^ Teff cell proliferation when compared to Treg cells from WT-mice (S2 Fig). To further study the role of FGL2 regarding Treg functions, spleen cells from AE-WT mice were exposed to either anti-FGL2-MAb or rFGL2. In response to rFGL2, Tregs from AE-WT mice inhibited ConA-induced CD4^+^ Teff proliferation; conversely, the same Tregs were not able to inhibit CD4^+^ Teff proliferation in the presence of anti-FGL2-MAbs (Fig 2F).

### T cell functions are less affected in *fgl2* ^*-/-*^ mice at the late stage of infection

**Fig 3:**
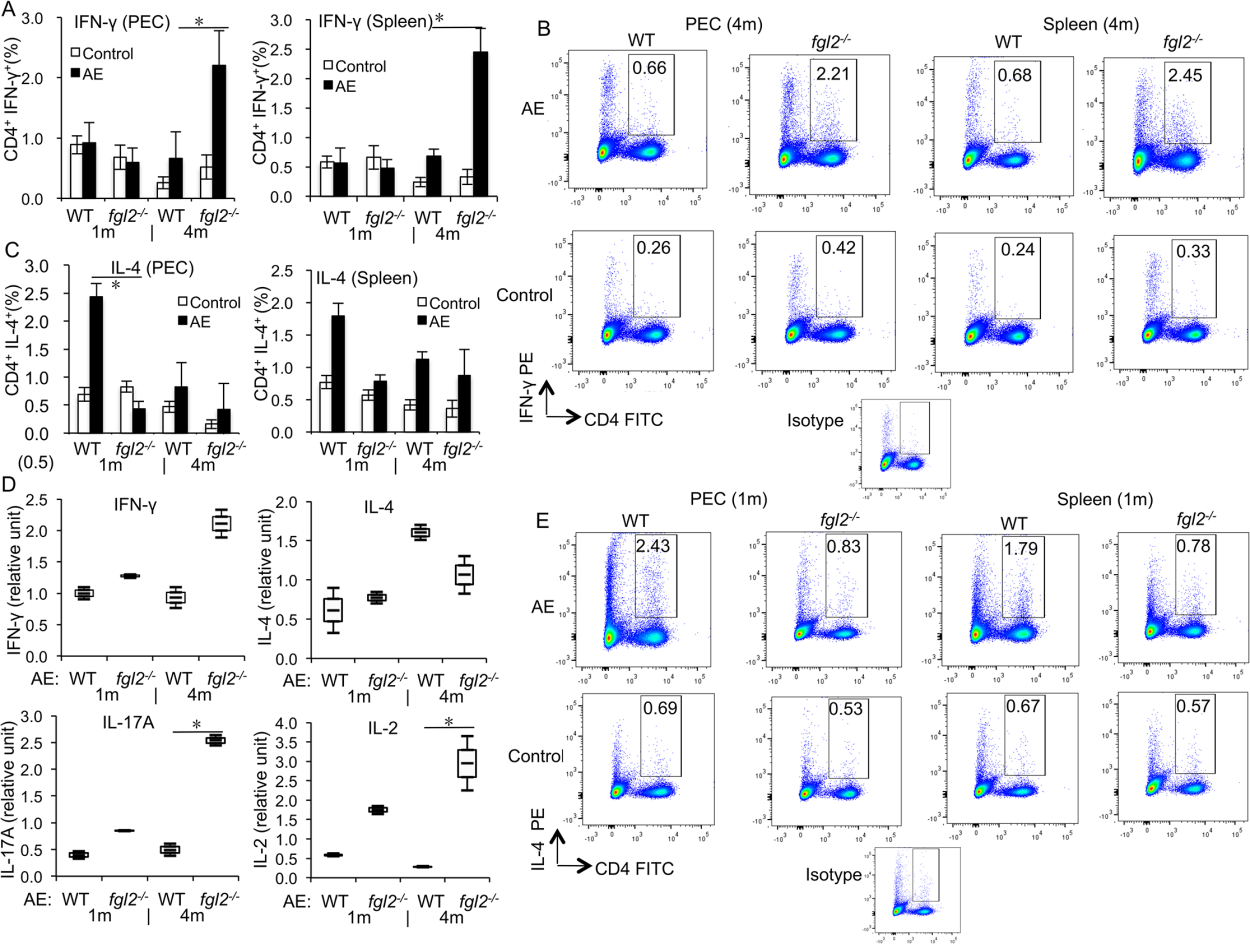
T cell function in both AE-WT and AE-*fgl2* ^*-/-*^ mice at 1 months and 4 months post-infection. (A) Frequency of IFN-γ^*+*^ T cells within CD4^*+*^ T cells in peritoneal and spleen cells from AE-WT and AE-*fgl2* ^*-/-*^ mice. (B) Representative images of IFN-γ^*+*^ T cells within CD4^*+*^ T cells in PECs and spleen cells from both AE-WT and AE-*fgl2* ^*-/-*^ mice at 4 months post-infection. (C) Frequency of IL-4^*+*^ T cells within CD4^*+*^ T cells in PECs and spleen cells from AE-WT and AE-*fgl2* ^*-/-*^ mice. (D) Relative level of T cell reactivity markers in spleen cells from AE-WT and AE-*fgl2* ^*-/-*^ mice, co-cultured with ConA (2 μg/mL), normalized with their corresponding non-infected controls (non-infected control was considered as baseline, e.g. 1.0). (E) Representative images of IL-4^*+*^ T cells within CD4^*+*^ T cells in PECs and spleen cells from both AE-WT and AE-*fgl2* ^*-/-*^ mice at 1 month post *E*. *multilocularis* infection. The corresponding isotype control antibodies were identically labeled as staining controls. Data represent mean±SD of three independent experiments of a total of 15–18 mice in each group (5–6 mice per group in each independent experiment). Comparison between groups was performed using a one-way ANOVA with Bonferroni’s multiple comparison post-test for statistical analysis. *P<0.025 for *ex vivo* assay, and *P<0.006 for *in vitro* assay. ‘WT’, wild type mice; ‘*fgl2* ^*-/-*^’, *fgl2* knock-out mice; ‘AE-WT’, *E*. *multilocularis*-infected wild type mice; ‘AE-*fgl2* ^*-/-*^’, *E*. *multilocularis*-infected *fgl2* knock-out mice. ‘PEC’, peritoneal exudate cells; ‘Spleen’, spleen cells.

To further explore the effects of FGL2 on the immune response during *E*. *multilocularis* infection, T cell functions, in AE-WT and AE-*fgl2* ^*-/-*^ mice were comparatively assessed. Purified splenic CD4^+^ T cells from AE-*fgl2* ^*-/-*^mice exhibited an increased proliferation in response to ConA, as compared to splenic CD4^+^ T cells from AE-WT mice (*P*<0.01) (S3A and S3B Fig). To further study the role of FGL2 in Tcell proliferation, splenic CD4^+^ T cells from WT mice were cultured in the presence and absence of rFGL2. CD4^+^ Teffs showed a pronounced proliferation in response to ConA stimulation, which was inhibited by the addition of rFGL2 (S3C Fig). Furthermore, T helper (Th) cells from AE-*fgl2* ^*-/-*^ mice appeared oriented towards a lower Th2 response at early stage of infection (1 months p.i.), and a stronger Th1-response at late stage of infection (4 months p.i.) (Fig 3A–3C and 3E). This dichotomic polarization was, however, not confirmed by *in vitro* cultivation of the cells in the presence of ConA stimulation (Fig 3D). Respective flow cytometric analyses of CD4^+^ T cells in spleen from AE-*fgl2* ^*-/-*^ mice and AE-WT mice showed that there was no difference in expression of IFN-γ and IL-4 at 48 h after of exposure to ConA (Fig 3D). However, expression levels of IL-17A and IL-2 were significantly higher in CD4^+^ T cells in spleen cell cultures obtained from AE-*fgl2* ^*-/-*^ mice at 4 months p.i. when compared to AE-WT mice.

### DC maturation is less affected in absence of FGL2 at the late stage of infection

**Fig 4:**
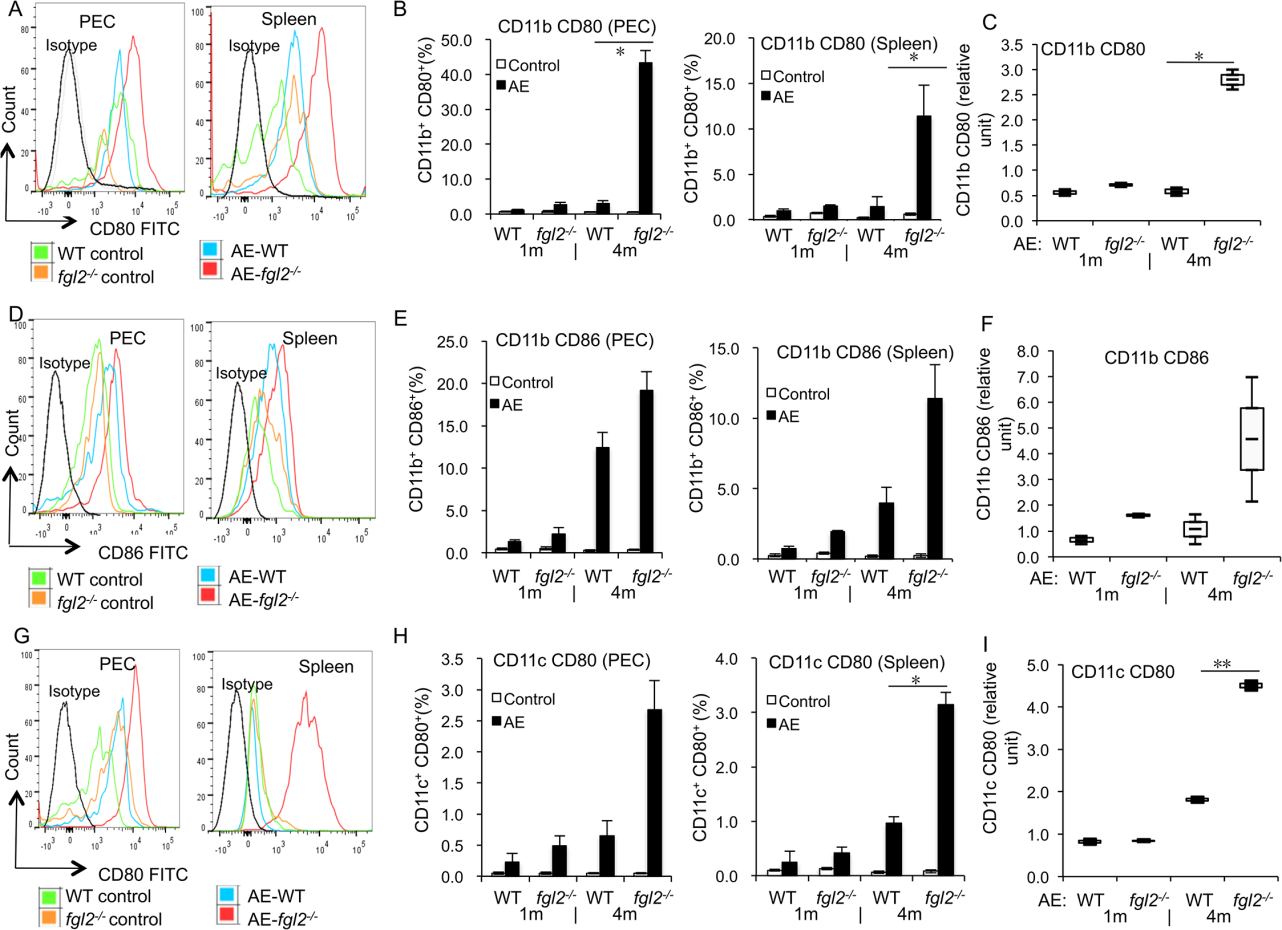
DC maturation in both AE-WT and AE-*fgl2* ^*-/-*^ mice at 1 month and 4 months post-infection. (A) Representive images of CD80 mean fluorescence intensity (MFI) in CD11b^*+*^ cells from AE-WT and AE-*fgl2* ^*-/-*^ mice, and non-infected mice as controls (4 months p.i.). (B) CD80 frequency within CD11b^*+*^ DCs in PECs and spleen cells of AE-WT and AE-*fgl2* ^*-/-*^ mice. (C) CD80 frequency within CD11b^*+*^ DCs in spleen cells from AE-WT and AE-*fgl2* ^*-/-*^ mice, co-cultured with ConA (2 μg/mL), normalized with their corresponding non-infected controls (we considered non-infected control as baseline, e.g. 1.0). (D) Representive images of CD86 MFI in CD11b^*+*^ cells from AE-WT and AE-*fgl2* ^*-/-*^ mice, and non-infected mice as controls (4 months p.i.). (E) CD86 frequency within CD11b^*+*^ DCs in PECs and spleen cells of AE-WT and AE-*fgl2* ^*-/-*^ mice. (F) CD86 frequency within CD11b^*+*^ DCs in spleen cells from AE-WT and AE-*fgl2* ^*-/-*^ mice, co-cultured with ConA (2 μg/mL), normalized with their corresponding non-infected controls (we considered non-infected control as baseline, e.g. 1.0). (G) Representive images of CD80 MFI in CD11c^*+*^ cells from AE-WT and AE-*fgl2* ^*-/-*^ mice, and non-infected mice as controls (4 months p.i.). (H) CD80 frequency within CD11c^*+*^ DCs in PECs and spleen cells of AE-WT and AE-*fgl2* ^*-/-*^ mice. (I) CD80 frequency within CD11c^*+*^ DCs in spleen cells from AE-WT and AE-*fgl2* ^*-/-*^ mice, co-cultured with ConA (2 μg/mL), normalized with their corresponding non-infected controls (non-infected control as baseline, e.g. 1.0). The corresponding isotype control antibodies were identically labeled for use as staining controls. Data represent mean±SD of three independent experiments of a total of 15–18 mice in each group (5–6 mice per group in each independent experiment). Comparison between groups was performed using a one-way ANOVA with Bonferroni’s multiple comparison post-test for statistical analysis. *P<0.01 for *ex vivo* assay, and *P<0.006 for *in vitro* assay. ‘WT’, wild type mice; ‘*fgl2* ^*-/-*^’, *fgl2* knock-out mice; ‘AE-WT’, *E*. *multilocularis*-infected wild type mice; ‘AE-*fgl2* ^*-/-*^’, *E*. *multilocularis*-infected *fgl2* knock-out mice. ‘PEC’, peritoneal exudate cells; ‘Spleen’, spleen cells.

The role of FGL2 in the maturation of different subsets of DCs, i.e. CD11b^+^ and CD11c^+^ DCs, was investigated. First, the maturation levels in both PECs and spleen cells from infected AE-*fgl2* ^*-/-*^mice, AE-WT mice, and non-infected controls were studied. Among CD11b^+^ DCs, the frequency of the maturation marker CD80 in PECs and spleen cells was higher at 4 months p.i. in AE-*fgl2* ^*-/-*^ mice than in AE-WT mice (Fig 4A and 4B). The same was found for CD11c^+^ DCs (Fig 4G and 4H). However, there was no difference in CD86 frequency in both subpopulations of DCs (Fig 4D and 4E). Subsequently, we assessed the same parameters, but upon *in vitro* cultivation and stimulation with ConA for 48 h. Findings revealed that CD80 frequency, not CD86, in both DC subpopulations from AE-*fgl2* ^*-/-*^ mice at 4 months p.i. was significantly higher than in those from AE-WT mice (Fig 4C,4F and 4I).

### *E*. *multilocularis* vesicle VF modulates T cell functions and DC maturation

**Fig 5:**
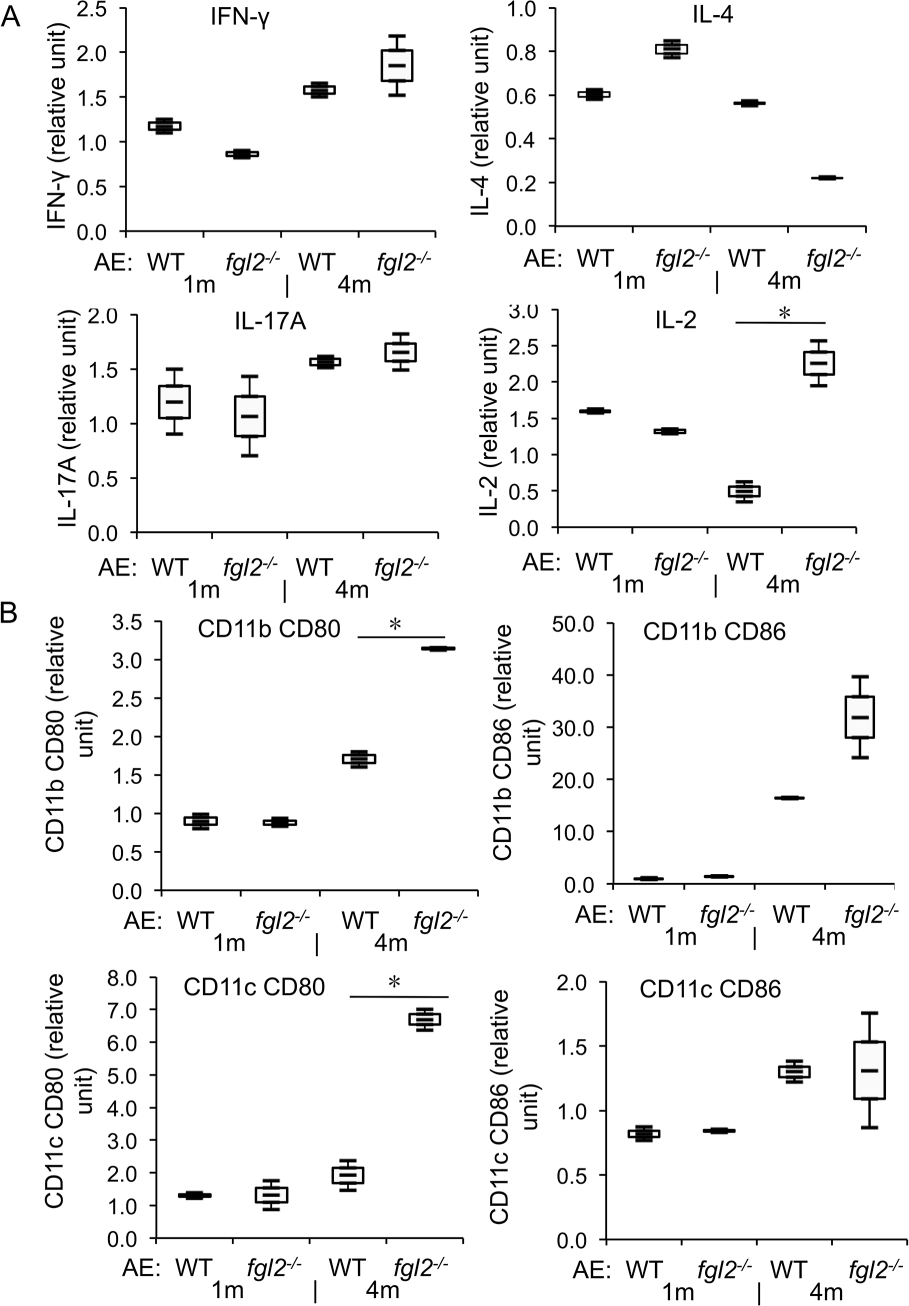
T cell reactivity and DC maturation in response to vesicle fluid (VF) in *E*. *multilocularis* infected mice at 1 month and 4 months p.i. (A) Relative expression level of T cell reactivity markers in spleen cells from AE-WT and AE-*fgl2* ^*-/-*^ mice, co-cultured with VF (10 μg/mL), in the presence of a protein transport inhibitor cocktail (Ebioscience, San Diego, CA, USA), normalized using cells from non-infected controls as baseline (e.g. 1.0). (B) Relative expression level of DC maturation markers in spleen cells from infected AE-WT and AE-*fgl2* ^*-/-*^ mice, co-cultured with VF (10 μg/mL), normalized using cells from non-infected controls. Data represent mean±SD of three independent experiments of a total of 15–18 mice in each group (5–6 mice per group in each independent experiment). Comparison between groups was performed using a one-way ANOVA with Bonferroni’s multiple comparison post-test for statistical analysis. *P<0.006. ‘WT’, wild type mice; *‘fgl2* ^*-/-*^’, *fgl2* knock-out mice; ‘AE-WT’, *E*. *multilocularis*-infected wild type mice; ‘AE-*fgl2* ^*-/-*^’, *E*. *multilocularis*-infected *fgl2* knock-out mice.

Flow cytometry of spleen cells exposed to VF for 96 h showed that the expression of IL-2 was significantly higher in CD4^+^ cells from AE-*fgl2* ^*-/-*^ mice obtained after 4 months p.i. than in the corresponding cell population isolated from AE-WT mice. However, there was no difference in expression of IFN-γ, IL-4 or IL-17A between CD4^+^ cells from AE-*fgl2* ^*-/-*^ mice and AE-WT mice (Fig 5A). To further explore the role of VF on Tregs and FGL2 secretion, respectively, spleen cells from AE-WT mice and non-infected WT controls were each cultured in the presence of VF (10 μg/mL). FGL2 levels in the supernatants were determined by ELISA. An identical experiment was performed with CD4^+^CD25^+^ Tregs and CD4^+^ Teffs instead of spleen cells, in the presence of APCs. No differences in FGL2 levels in supernatants of sorted AE-WT Tregs, nor in CD4^+^ Teffs, could be detected in response to VF, when compared to cultures from non-infected animals (S4 Fig).

For DCs at 96 h after exposure to VF, the CD80 frequency, both in CD11b^+^ and CD11c^+^ DCs from AE-*fgl2* ^*-/-*^ mice at 4 months p.i., was significantly higher than in DCs from AE-WT mice. However, there was no difference in CD86 frequency in both subpopulations of DCs from AE-*fgl2* ^*-/-*^ mice after exposure to VF, compared to DCs from AE-WT mice (Fig 5B).

### Recombinant FGL2 and anti-FGL2–MAbs affect T cell functions, co-stimulation and DC maturation in spleen cells from non-infected WT mice

**Fig 6:**
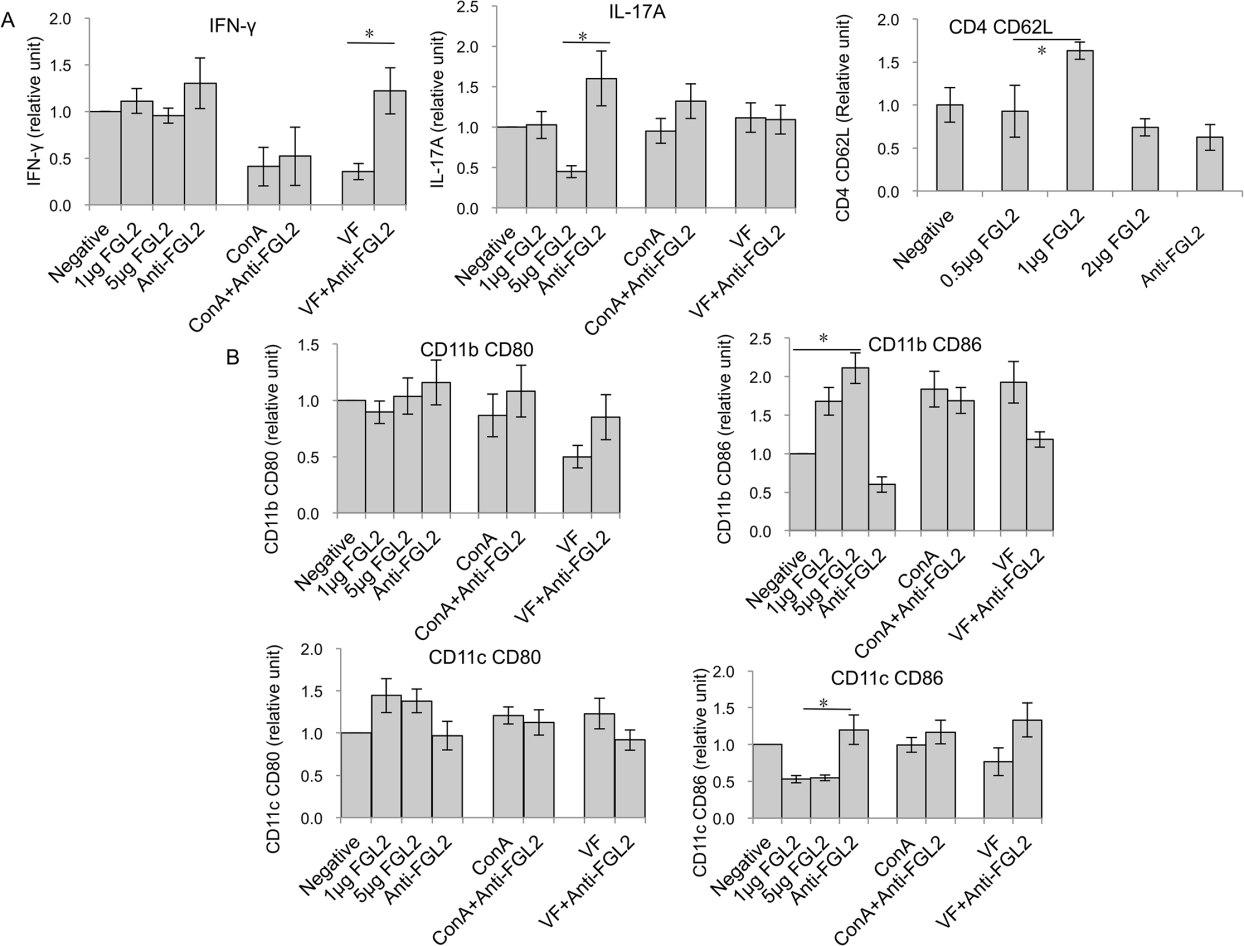
Recombinant FGL2 induces down regulation of T cell reactivity and DC maturation *in vitro*. Different concentrations of recombinant FGL2 (0, 1, 5 μg/mL), ConA or vesicle fluid (VF), and anti-FGL2-MAb (1 μg/mL) were added to primary spleen cells isolated from non-infected WT mice and cultured in the presence of protein transport inhibitor cocktail. Relative expression level of T cell reactivity (A) and DC maturation markers (B) were determined by flow cytometry. Data represent mean±SD of three independent experiments of a total of 15–18 mice in each group (5–6 mice per group in each independent experiment). Expression of each maker was normalized with cells without rFGL2 and anti-FGL2-MAb (negative control) as base line (e.g. as 1.0). Comparison between groups was performed using a one-way ANOVA with Bonferroni’s multiple comparison post-test for statistical analysis. *P<0.006.

To further confirm the role of FGL2 on T cell functions and DC maturation, spleen cells from non-infected WT mice were cultured in the presence or absence of rFGL2 or anti-FGL2-MAbs. Flow cytometry showed that IFN-γ expression was specifically increased in response to VF in the presence of anti-FGL2 MAbs, and IL-17A expression was decreased in the presence of high concentration of rFGL2 (5 μg/mL) and there was no influence on IL-17 expression upon exposure to anti-FGL2-MAb (Fig 6A). The expression of CD62L, a cell adhesion molecule that is abundant on naïve lymphocytes, but not expressed on effector memory T-lymphocytes [29], was found to be increased on CD4^+^ T cells in the presence of rFGL2 (1 μg/mL), but decreased in the presence of anti-FGL2 antibodies (Fig 6A). However, this was not the case upon analysis of total spleen cell populations (S5 Fig), which indicated that FGL2 might play an important role in down-regulating lymphocyte co-stimulation and effector T cell production.

For DCs, the expression of CD86 on CD11c^+^ DCs was decreased in the presence of rFGL2, but increased in the presence of anti-FGL2 MAbs. However, expression of CD86 on CD11b^+^ DCs showed the opposite (Fig 6B).

## Discussion

During infection with *E*. *multilocularis* metacestodes, immune tolerance and/or down-regulation of immunity is a marked characteristic that becomes more pronounced at the later stage of chronic disease in both humans [30] and in experimentally infected mice [5]. In the present study, we identified FGL2 as an important mediator of susceptibility to *E*. *multilocularis* infection in mice, and demonstrated for the first time that FGL2 a) partially contributes to Treg functions; b) has the capacity to down-regulate the maturation of DCs; c) suppresses Th1- and Th17-type immune responses; d) promotes Th2-biased and Treg immune responses; and finally that e) IL-17A contributes to FGL2-secretion.

Earlier microarray-based data obtained from *E*. *multilocularis*-infected liver tissue have shown that the peri-parasitic area is characterized by an increased FGL2 expression [7]. Since immunomodulation is a hallmark of AE, and Treg cells are one of the key immune subsets to mediate this effect, our objective was to study the role of Treg-expressed FGL2 in the outcome of *E*. *multilocularis* infection. We originally hypothesized that upregulated FGL2-expression at the host-parasite interface promotes parasite proliferation. Comparative analysis of *E*. *multilocularis* growth in *fgl2* ^*-/-*^ mice versus WT mice at the late stage of infection showed clearly that AE-WT mice exhibited a significantly higher parasite load as compared to AE-*fgl2* ^*-/-*^ mice. In parallel, the parasite proliferation potential, assessed by determination of the *E*. *multilocularis em14-3-3* gene-expression-level, was significantly higher in AE-WT mice, and respectively lower in AE-*fgl2* ^*-/-*^ animals. In previous experiments we had already documented that *em14-3-3*-expression significantly increased upon reduced protective immunity such as encountered in athymic nude mice, or decreased when the parasite growth was hindered as e.g. by albendazole therapy [28,31]. AE-WT-mice also exhibited higher FGL2-levels in the serum as compared to non-infected mice, elevated *fgl*2 mRNA expression levels in respective PEC and spleen cells, and a concomitantly increased number of Tregs, which represent one of the major Treg producers [9]. Taken together, these results support the hypothesis that Treg-expressed FGL2 contributes to the pathogenesis of *E*. *multilocularis* infection. In other infection models, such as viral MHV-3 infection [10], similar results have been demonstrated, while no such effects were found during protozoan (*Toxoplasma gondii*), bacterial (*Yersinia enterocolitica*, *Listeria monocytogenes*, and *Mycobacterium tuberculosis*), and other types of viral infections such as murine gamma-herpesvirus-68 and Sendai infections [32]. The successful survival of pathogens depends mainly on evading the host immune response by, for example, varying their surface antigens, eliminating their protein coat, and/or modulating the host immune response. Immunosuppression is sometimes directly caused by pathogen-derived metabolic products, and sometimes involves antigenic mimicry. One of the most sophisticated mechanisms of immune evasion is the selective activation of a directly targeted subset of T helper cells. Different pathogens may target different regulative pathways of the host immune response, such as Th2, Tregs or Th17. It is known that the regulation of such pathways is not dependent on single genes or molecules. Rather, a complex immuno-network is usually involved, which requires a sophisticated modulatory process via specific modulatory molecules from *E*. *multilocularis* to become effective, some may even act in combination or exert together a synergistic effect. Already know *E*. *multilocularis* bioactive molecules include e.g. Alkaline Phosphatase [33], Em492 [34], EmTIP [7], among others; if these are involved in e.g. FGL2-induction needs to be further explored.

How FGL2 influences and/or modulates the outcome of experimental AE is still largely unclear, although some of our study data provide first indications. In experimental AE [5,35,36], the control of metacestode proliferation appears to be predominantly T cell-dependent, as assessed upon use of different immune-compromised mouse models [27,31,37], and confirmed by observations in human AE-patients with immune suppression-associated conditions [38–41]. It is therefore conceivable that the proliferating metacestode itself specifically activates and concurrently modulates the immune response to its own advantage. The metacestode appears to secrete immuno-active metabolites to suppress the protective Th1-type response, and to promote a secondarily reinforced Th2-type response through functional induction and maintenance of Treg cells [2, 25]. Tregs, which over-express a subset of regulatory cytokine genes including those coding for IL-10 and TGF-β, play an important role in promoting immune tolerance in a number of parasitic disease models [42]. In AE, the expression of both cytokines appears up-regulated in mice as shown by different studies [39], and other studies strongly suggested that they are also up-regulated in human AE-patients [43].

Various molecular and cellular mechanisms have been proposed to explain how Tregs suppress immune responses. These include cell-to-cell contact-dependent suppression, cytotoxicity, and immunoregulatory cytokine secretion such as IL-10 and TGF-β [44]. However, the importance of these cytokines remains controversial, as several reports have demonstrated that antibodies against IL-10 and TGF-β failed to block Treg suppressive function, and Tregs from TGF-β–deficient mice retained normal suppressive activity *in vitro* [44]. In addition, the ambiguous role of TGF-β, which is both, a strong inducer of immune tolerance and an activator of the pro-inflammatory IL-17 cytokine system, remains puzzling [45,46].

Undoubtedly, FGL2 represents an alternative candidate that could, at least partially, support regulatory, and thus immunosuppressive, functions of Tregs. In Tregs of WT BALB/c mice, FGL2 encoding mRNA is constitutively expressed at a high level, and expression even increased after MHV-3 infection, and adoptive transfer of WT Tregs into MHV-3 resistant *fgl2* ^*-/-*^ mice suggested that FGL2 might be an important Treg effector molecule [10]. Previous studies on FGL2 had shown that rFGL2 suppressed T cell proliferation induced by anti-CD3/28 MAbs and ConA [47,48]. In this study, we now demonstrated that rFGL2 suppressed CD4^+^ effector T cell proliferation in response to *E*. *multilocularis* antigenic metabolites present in the metacestode VF. We also showed that FGL2 inhibited the maturation of DCs, suppressed Th1 and Th17 immune responses, and FGL2 polarized an allogeneic immune response towards a Th2-oriented cytokine profile, both *in vivo* and *in vitro*. Conversely, in *fgl2* ^*-/-*^mice, Th1 cytokine levels and activity of DCs and T cells were all increased when compared to WT-animals, and FGL2-serum-levels correlated with IL-4 expression in WT-mice before and after *E*. *multilocularis* infection. The development of a Th2-oriented immune response in WT-mice during the course of *E*. *multilocularis* infection corroborated the generally known effect of FGL2 to promote a Th2 cytokine production, with a concomitant inhibition of Th1- and Th17-oriented immunity [47]. Furthermore, increased serum levels of IL-17A correlated with high FGL2 serum levels, suggesting for the first time that IL-17A could contribute to FGL2-secretion. This was confirmed *in vitro* by the finding that recombinant IL-17A promoted the production of FGL2 in spleen cells.

**Fig 7:**
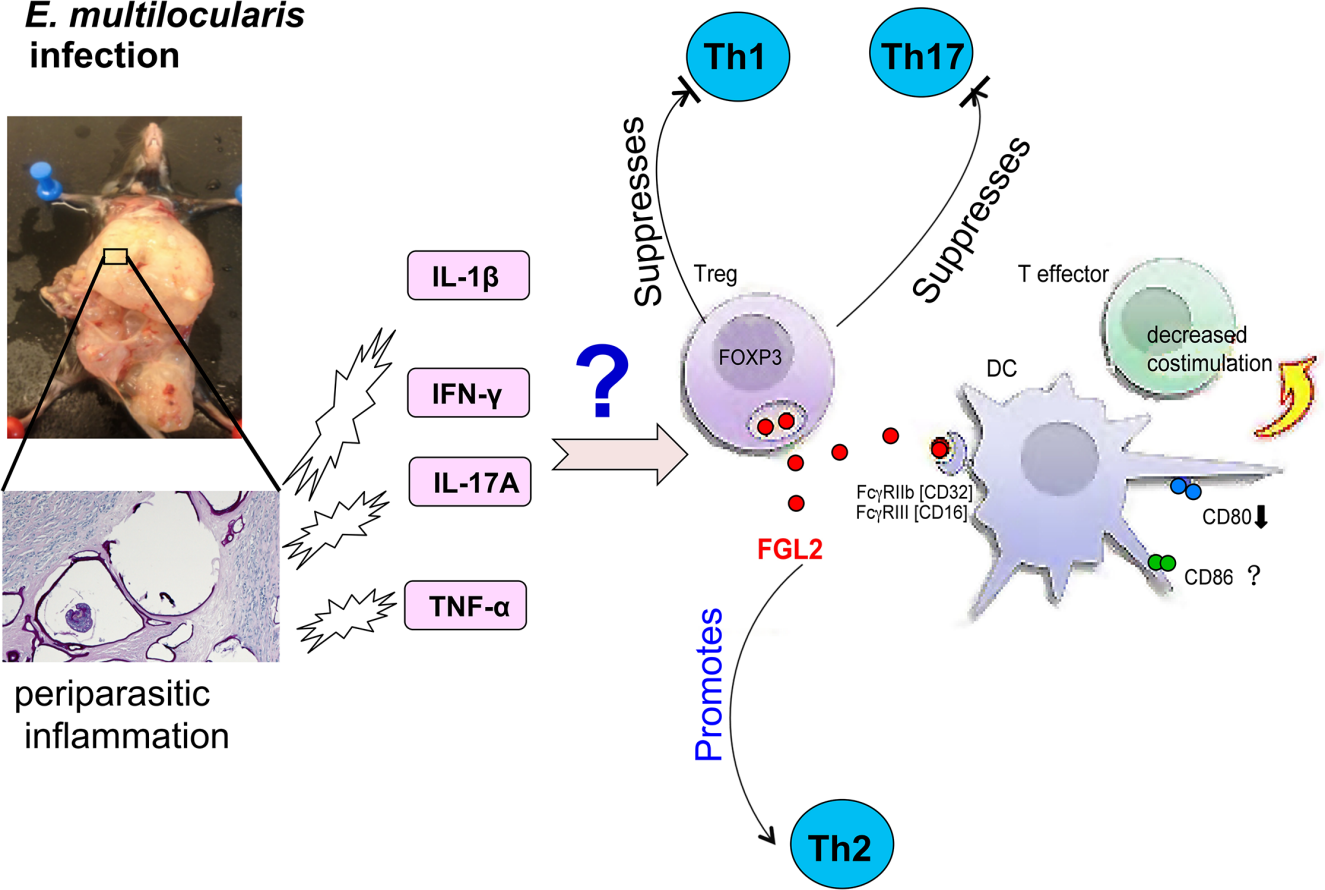
A schematic presentation of the hypothetical role of FGL2 in immune regulation during AE: *E*. *multilocularis* metabolites induce the release of TNF-α, IFN-γ and IL-17; IFN-γ as demonstrated previously, but also IL-17A as presently demonstrated, contributes to FGL2 secretion by Tregs and other cells; once FGL2 is released, it binds to FcγRIIB receptors, down-regulates the maturation of DCs, decreases co-stimulation of effector T cells, suppresses Th1 and Th17 immune response, accelerates Th2 immune responses, and overall this leads to an immune suppressed status that favors the continuous “tumor-like” progression of the parasitic metacestode.

We also investigated whether FGL2 would affect the functional activities of Treg cells, by directly assessing the effects of rFGL2 and of an anti-FGL2-MAb on Treg activities *in vitro*. The presence of recombinant FGL2 promoted Treg function, while addition of anti-FGL2-MAb completely abrogated Treg activity. Further evidence for the role of FGL2 as a molecule that promotes Treg function, and thus immunosuppression, was given by the observation that *fgl*2^-/-^ mice exhibited both decreased Treg numbers and impaired Treg function. The mechanism by which FGL2 mediates its immunosuppressive activity is currently under investigation. Previous studies have indicated that FGL2 binds to the inhibitory FcγRIIB receptor (CD32) expressed primarily on APCs. Both CD80 and CD86 ligands are found on APCs and are known to provide efficient costimulation, however, in our experiments, distinct functions may be attributed to CD80 and CD86. The differential functions of these molecules have already been the subject of considerable studies, with most data suggesting that the two ligands share substantially overlapping functions. There is, however, also an increasing evidence that supports the view that CD80 may be a more effective ligand for CTLA-4 than CD86 [49]. This FGL2-FcγRIIB interaction through CD80 was shown to induce apoptosis in B cells, and to inhibit DC-maturation [11]. In *E*. *multilocularis* infection, several immune subsets may express the FcγRIIB receptor, such as macrophages (including the ‘epithelioid cells’ that line the ‘immuno-modulating’ laminated layer), and also the numerous CD8^+^ T cells present in the periparasitic infiltrate [50]; CD8^+^ T cells have actually been shown to express the FcγRIIB receptor in a murine model of *Trypanosoma cruzi* [51]. Taken our recent data on the course of cytokine expression by the periparasitic immune infiltrate in *E*. *multilocularis* infection [4] combined with the results from this study we propose that in *E*. *multilocularis* infected mice the following events occur: (a) TNF-α, IFN-γ and IL-17A are released by the host at early stage of infection; (b) these cytokines, and especially IFN-γ as demonstrated previously [32] but also IL-17A as shown in the present study, contribute to FGL2-secretion by Tregs and other cells; and (c) secreted FGL2 can bind to FcγRIIB receptor, leads to a late-stage immune suppression by down-regulating the maturation of DCs, decreases co-stimulation of effector T cells, suppresses Th1 and Th17 immune responses, and accelerates Th2 immune responses. Some parasite's specific molecules may be involved in the modulation of FGL2, as discussed earlier. Overall, this will lead to a periparasitic immune suppressed (anergic) status that favors the continuous “tumor-like” progression of the parasite (Fig 7). Direct inhibition of macrophage and/or mast cell functions could also be induced by this interaction [24].

These findings on the role of FGL2 in *E*. *multilocularis* infection now open the door for more applied approaches. For instance, the experimental treatment of *E*. *multilocularis* infected mice with anti-FGL2-MAb could provide a means of converting the immunological anergy during chronic disease into a more pro-active, Th1-oriented immunity, with potentially fatal consequences for the metacestode. This is already under investigation. Furthermore, our findings may provide a rationale for studying FGL2 as a target for an immunomodulatory treatment option in patients with progressive AE. In addition, FGL2 may be proposed also as a serum marker indicative for progression of *E*. *multilocularis* infection, may be useful to assess the clinical status of AE-patients and the course and outcome and/or parasite activity in human AE.

## Supporting Information

S1 Fig Gating strategy for the assessment of different cytokines and Foxp3. (A) Gating strategy for the assessment of different cytokines: gate (G1) was positioned around lymphocytes, and cells within this gate were used for identifying CD4^+^ T cells. Cytokine frequency was based on the fluorescence signal of different cytokine stainings; (B) Gating strategy for the assessment of different cytokines: gate (G1) was positioned around lymphocytes, and cells within this gate were used for identifying CD4^+^ T cells. Based on the fluorescence signal of the CD25 staining, CD25^+^ cells with bright fluorescence signal were distinguished as CD4^+^CD25^+^ cells (G2). Within gate G2, Foxp3 histogram plots were used to determine the number of Foxp3^+^ cells.
(TIF)

S2 Fig Treg suppression assay. By using MACS and a followed FACS, CD4^+^CD25^-^ Teff cells (responders) were isolated from spleen cells of non-infected WT mice, CD4^+^CD25^+^ Tregs (suppressor cells) were isolated from spleen cells of Control-WT mice (A), AE-WT mice (B), Control- *fgl2* ^*-/-*^ mice (C), AE- *fgl2* ^*-/-*^ mice (D). CD4^+^CD25^+^ Tregs (suppressor cells) and CD4^+^CD25^-^ Teff cells (responder cells) were co-cultured at different suppressor: responder ratios in the presence of syngeneic APCs and anti-CD3 antibody (0.5 μg/mL). Cell proliferation was measured using BrdU ELISA. ‘WT’, wild type mice; ‘*fgl2* ^*-/-*^’, *fgl2* knock-out mice; ‘AE-WT’, *E*. *multilocularis*-infected wild type mice; ‘AE-*fgl2* ^*-/-*^’, *E*. *multilocularis*-infected *fgl2* knock-out mice.
(TIF)

S3 Fig CD4^+^ T cell proliferation in WT and *fgl2* ^*-/-*^ mice at 4 months post *E*. *multilocularis* infection. CD4^+^ T cells (Teffs) in the presence of APCs (A) or spleen cells (B) from AE-WT and AE-*fgl2* ^*-/-*^ mice were cultured with ConA (2 μg/mL) for 48h. The presented data were normalized with their own non-infected controls for statistical analyses (we considered non-infected control as baseline, e.g. as 1.0). (C) Different concentrations of recombinant FGL2 (0, 1, 5 μg/mL), ConA or vesicle fluid (VF), and anti-FGL2-MAb (1 μg/mL) were added to primary spleen cells isolated from AE-WT mice, compared to cultures from non-infected animals. CD4^+^ T cell proliferation was determined by BrdU ELISA. Comparison between groups was performed using a one-way ANOVA. *P<0.05.
(TIF)

S4 Fig FGL2 levels in supernatants from Tregs and Teffs cultures in the presence of APCs. Experiments include WT and *fgl2* ^*-/-*^ mice at 4 months post *E*. *multilocularis* infection. Tregs and CD4^+^ T cells (Teffs) in the presence of APCs from AE-WT and Control-WT mice were cultured with VF (10 μg/mL) for 96h. FGL2-levels (culture supernatants) were determined by ELISA. Data represent mean±SD of three independent experiments of a total of 15–18 mice for each group (5–6 mice per group in each independent experiment). Comparison between groups was performed using a one-way ANOVA. *P<0.05.
(TIF)

S5 Fig CD62L-expression in response to rFGL2 and anti-FGL2. Different concentrations of recombinant FGL2 (0, 1, 2 μg/mL) and anti-FGL2-MAb (1 μg/mL), and PMA as a positive control, were added to primary spleen cells isolated from non-infected WT mice. CD62L-percentage in total spleen cells was determined by flow cytometry. Data represent mean±SD of three independent experiments of a total of 15–18 mice for each group (5–6 mice per group in each independent experiment). Comparison between groups was performed using a one-way ANOVA *P<0.05.
(TIF)
