# Supplementary figures and images for: Deletion of Fibrinogen-like Protein 2 (FGL-2), a Novel CD4+ CD25+ Treg Effector Molecule, Leads to Improved Control of Echinococcus multilocularis Infection in Mice
Source: PLoS Negl Trop Dis. 2015 May 8;9(5):e0003755. doi: 10.1371/journal.pntd.0003755 (PMC4425495; doi:10.1371/journal.pntd.0003755)

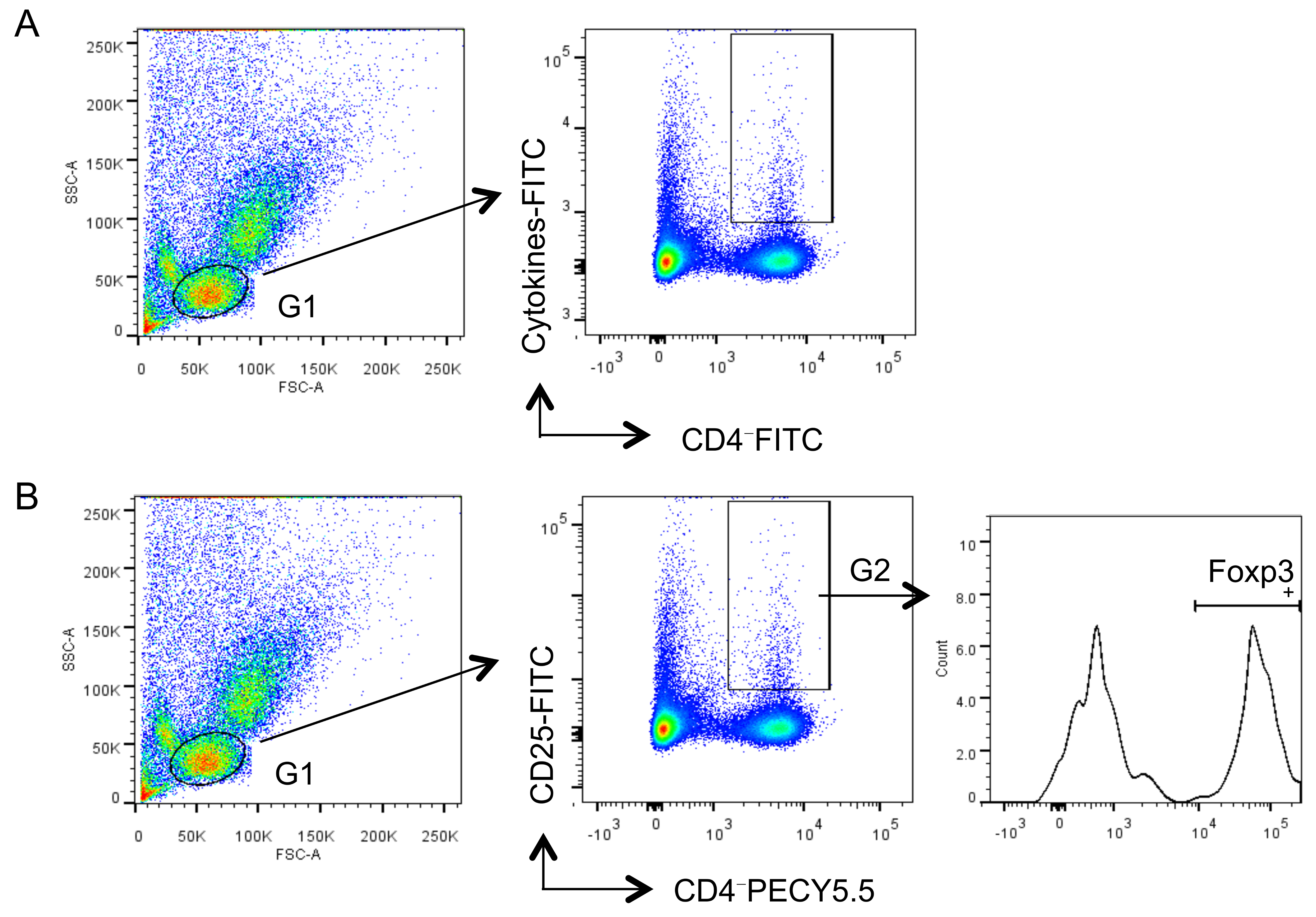

Supplement: S1 Fig — (A) Gating strategy for the assessment of different cytokines: gate (G1) was positioned around lymphocytes, and cells within this gate were used for identifying CD4+ T cells. Cytokine frequency was based on the fluorescence signal of different cytokine stainings; (B) Gating strategy for the assessment of different cytokines: gate (G1) was positioned around lymphocytes, and cells within this gate were used for identifying CD4+ T cells. Based on the fluorescence signal of the CD25 staining, CD25+ cells with bright fluorescence signal were distinguished as CD4+CD25+ cells (G2). Within gate G2, Foxp3 histogram plots were used to determine the number of Foxp3+ cells. (TIF) [file pntd.0003755.s001.tif]

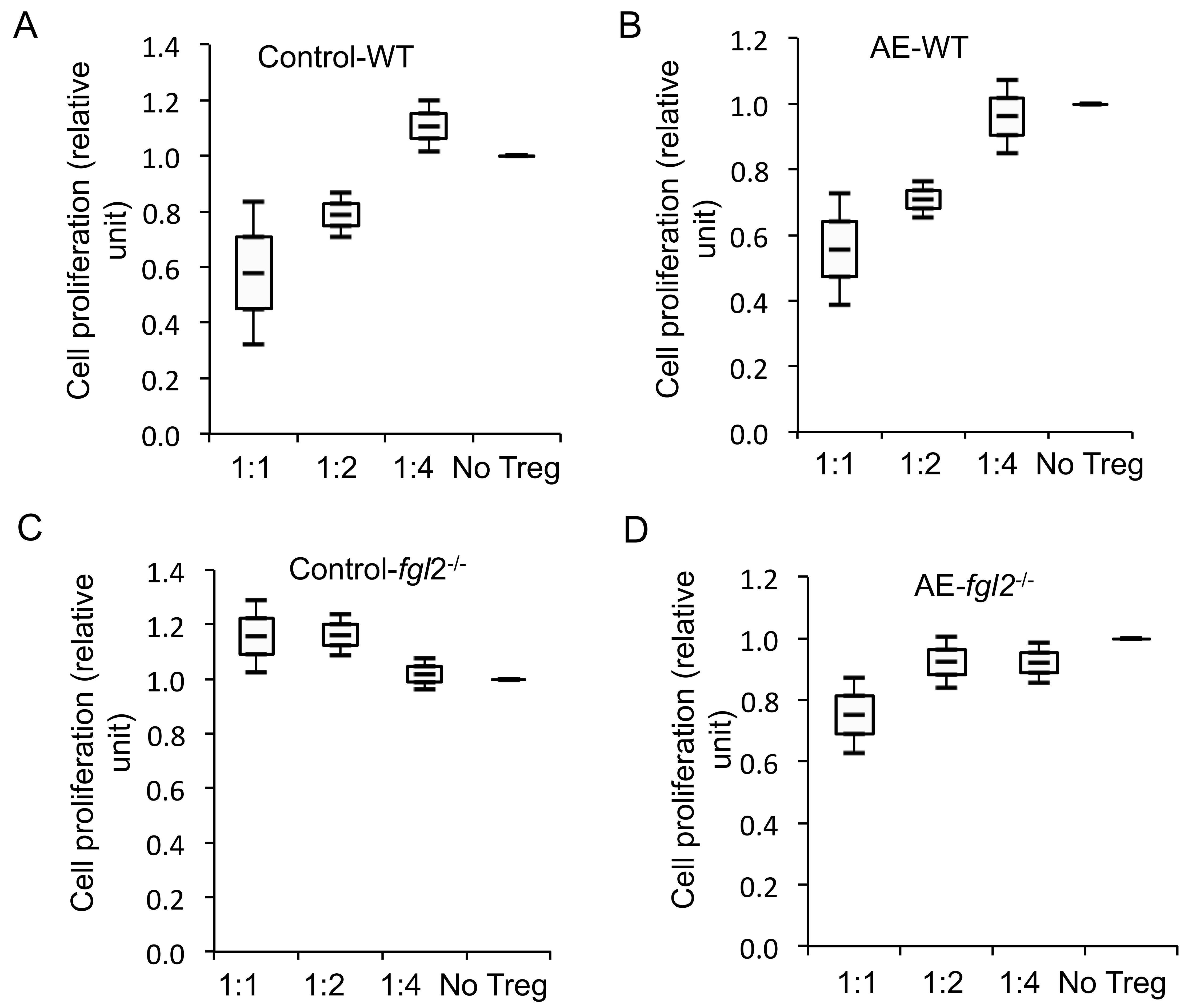

Supplement: S2 Fig — By using MACS and a followed FACS, CD4+CD25- Teff cells (responders) were isolated from spleen cells of non-infected WT mice, CD4+CD25+ Tregs (suppressor cells) were isolated from spleen cells of Control-WT mice (A), AE-WT mice (B), Control- fgl2 -/- mice (C), AE- fgl2 -/- mice (D). CD4+CD25+ Tregs (suppressor cells) and CD4+CD25- Teff cells (responder cells) were co-cultured at different suppressor: responder ratios in the presence of syngeneic APCs and anti-CD3 antibody (0.5 μg/mL). Cell proliferation was measured using BrdU ELISA. ‘WT’, wild type mice; ‘fgl2 -/-’, fgl2 knock-out mice; ‘AE-WT’, E. multilocularis-infected wild type mice; ‘AE-fgl2 -/-’, E. multilocularis-infected fgl2 knock-out mice. (TIF) [file pntd.0003755.s002.tif]

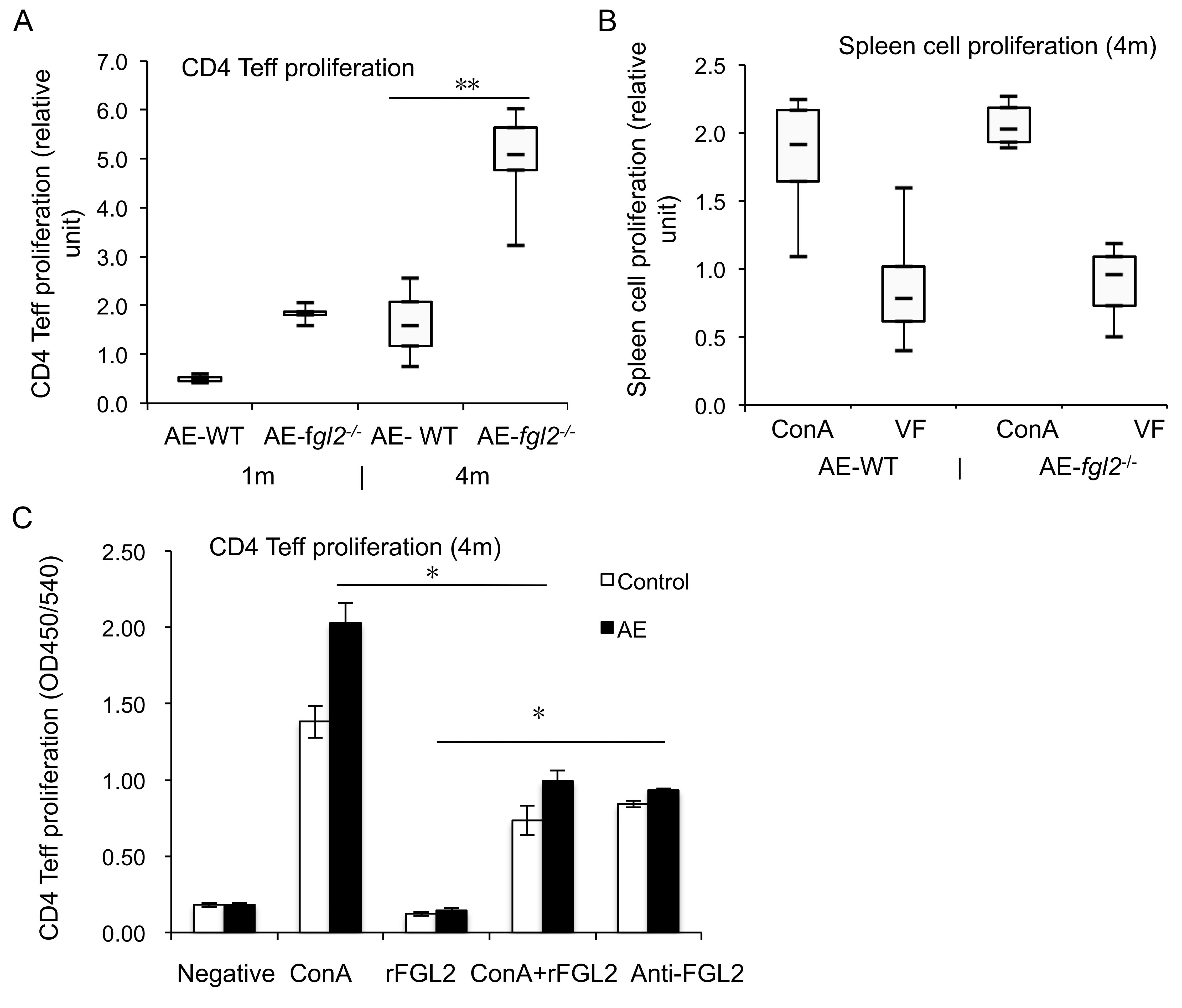

Supplement: S3 Fig — CD4+ T cells (Teffs) in the presence of APCs (A) or spleen cells (B) from AE-WT and AE-fgl2 -/- mice were cultured with ConA (2 μg/mL) for 48h. The presented data were normalized with their own non-infected controls for statistical analyses (we considered non-infected control as baseline, e.g. as 1.0). (C) Different concentrations of recombinant FGL2 (0, 1, 5 μg/mL), ConA or vesicle fluid (VF), and anti-FGL2-MAb (1 μg/mL) were added to primary spleen cells isolated from AE-WT mice, compared to cultures from non-infected animals. CD4+ T cell proliferation was determined by BrdU ELISA. Comparison between groups was performed using a one-way ANOVA. *P<0.05. (TIF) [file pntd.0003755.s003.tif]

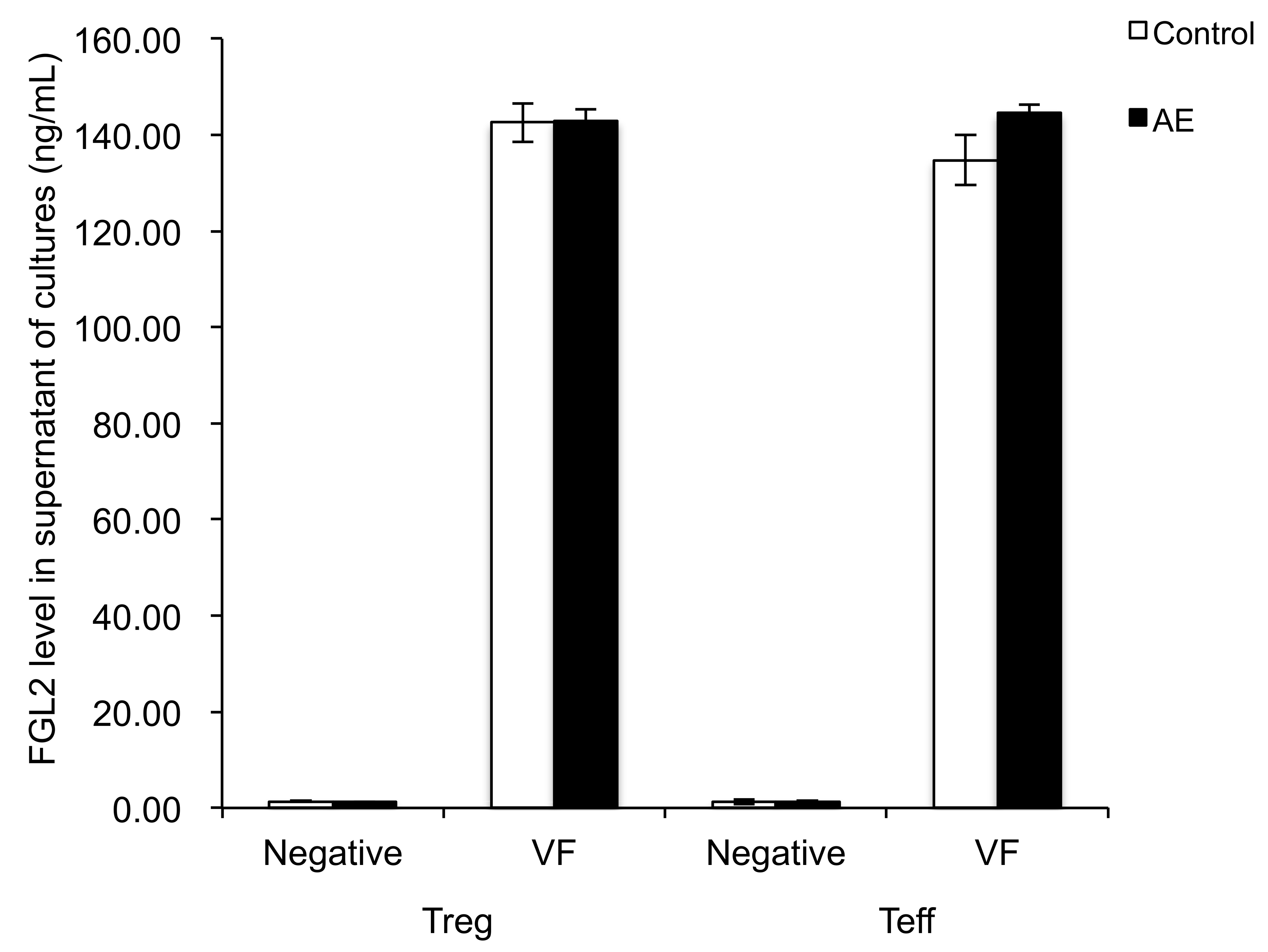

Supplement: S4 Fig — Experiments include WT and fgl2 -/- mice at 4 months post E. multilocularis infection. Tregs and CD4+ T cells (Teffs) in the presence of APCs from AE-WT and Control-WT mice were cultured with VF (10 μg/mL) for 96h. FGL2-levels (culture supernatants) were determined by ELISA. Data represent mean±SD of three independent experiments of a total of 15–18 mice for each group (5–6 mice per group in each independent experiment). Comparison between groups was performed using a one-way ANOVA. *P<0.05. (TIF) [file pntd.0003755.s004.tif]

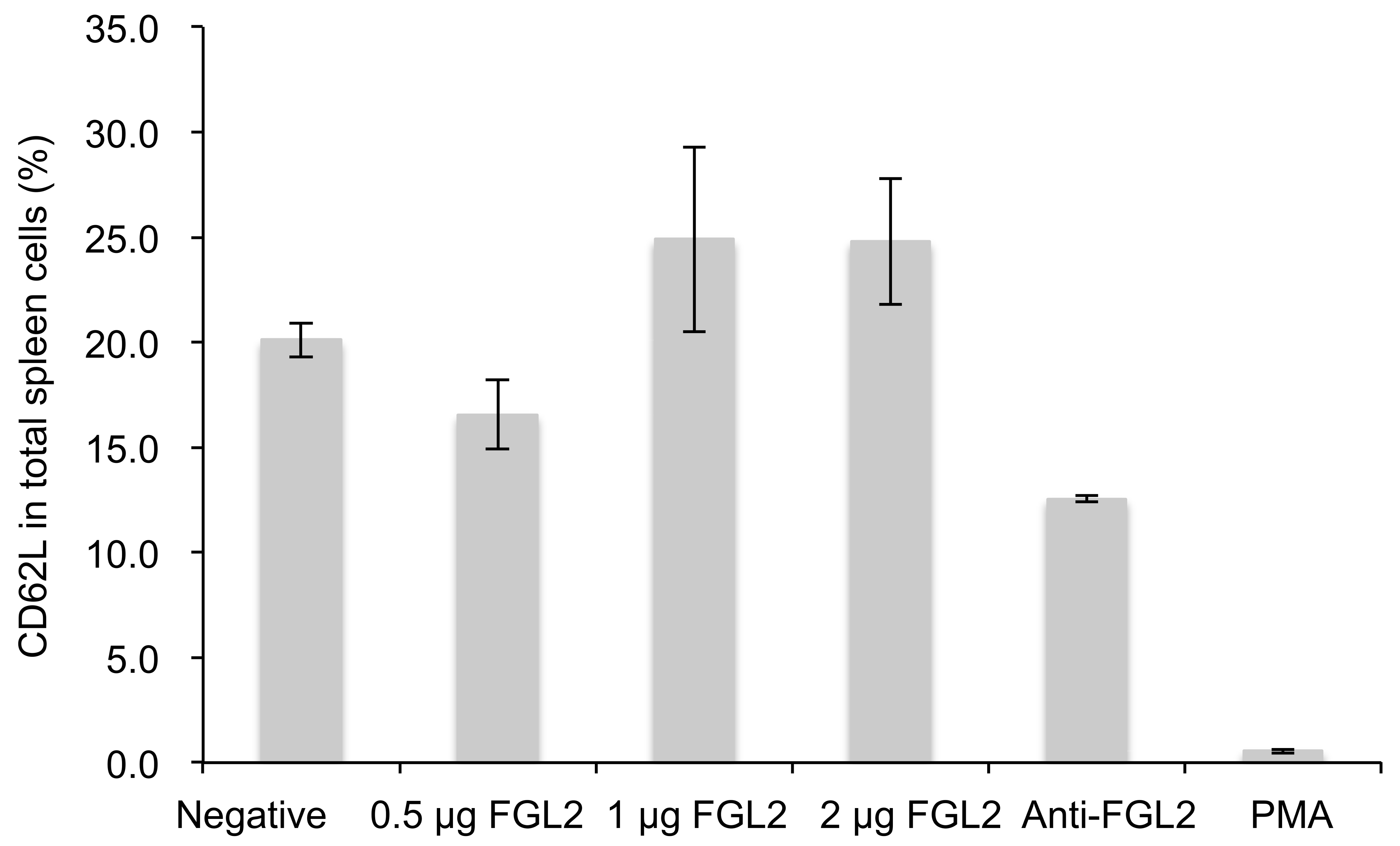

Supplement: S5 Fig — Different concentrations of recombinant FGL2 (0, 1, 2 μg/mL) and anti-FGL2-MAb (1 μg/mL), and PMA as a positive control, were added to primary spleen cells isolated from non-infected WT mice. CD62L-percentage in total spleen cells was determined by flow cytometry. Data represent mean±SD of three independent experiments of a total of 15–18 mice for each group (5–6 mice per group in each independent experiment). Comparison between groups was performed using a one-way ANOVA *P<0.05. (TIF) [file pntd.0003755.s005.tif]
